# Green Tea Polyphenols for the Protection against Renal Damage Caused by Oxidative Stress

**DOI:** 10.1155/2012/845917

**Published:** 2012-07-10

**Authors:** Takako Yokozawa, Jeong Sook Noh, Chan Hum Park

**Affiliations:** ^1^Institute of Natural Medicine, University of Toyama, Toyama 930-0194, Japan; ^2^Organization for Promotion of Regional Collaboration, University of Toyama, Toyama 930-8555, Japan

## Abstract

Green tea, prepared from the leaves of *Camellia sinensis* L., is a beverage that is popular worldwide. Polyphenols in green tea have been receiving much attention as potential compounds for the maintenance of human health due to their varied biological activity and low toxicity. In particular, the contribution of antioxidant activity to the prevention of diseases caused by oxidative stress has been focused upon. Therefore, in this study, we investigated the effects of (−)-epigallocatechin 3-*O*-gallate and (−)-epigallocatechin 3-*O*-gallate, which account for a large fraction of the components of green tea polyphenol, on oxidative stress-related renal disease. Our observations suggest that green tea polyphenols have a beneficial effect on pathological states related to oxidative stress of the kidney.

## 1. Background

Clinical and experimental studies have resulted in extensive discussions of the link between renal disease and oxidative stress, which is directly or indirectly derived from various pathological conditions such as hyperglycemia, free radical-generating toxic substances, and inflammation. The free radicals are highly reactive and harmful to lipids, proteins, and nucleic acids, resulting in structural and functional impairment. Increased levels of endproducts mediated by the reactions between biomolecules and free radicals, such as malondialdehyde, 3-nitrotyrosine, and 8-hydroxy-2′-deoxyguanosine, were observed with various pathological phenomena, such as acute renal failure and hemodialysis [1–4]. Inhibitors of free radicals and antioxidants have also been shown to protect against renal damage in a number of studies [5].

 Green tea polyphenols have been shown to act as metal chelators, preventing the metal-catalyzed formation of radical species, antioxidant enzyme modulators, and scavengers of free radicals, including the hydroxyl radical (∙OH), superoxide anion (O_2_^−^), nitric oxide (NO), and peroxynitrite (ONOO^−^) [6–12]. These antioxidant activities are considered to be closely related to their protective effects against various diseases, including renal disease, arteriosclerosis, cancer, and inflammation caused by lipid peroxidation and excessive free radical production [13]. The polyphenolic compounds of green tea mainly comprise (−)-epigallocatechin 3-*O*-gallate, (−)-epicatechin 3-*O*-gallate, (−)-epigallocatechin, and (−)-epicatechin, which are classified as the flavan-3-ol class of flavonoids. This paper gives a review of our recent findings [14–16], with emphasis on the therapeutic potential of the polyphenols of green tea in a useful experimental model of renal damage.

## 2. (−)-Epicatechin 3-*O*-gallate and ONOO^−^-Mediated Renal Damage

Evidence for the role of reactive oxygen and nitrogen metabolites in the pathogenesis of renal diseases has accumulated, and ONOO^−^ formed in vivo from O_2_^−^ and NO has been suggested to be an important causative agent in the pathogenesis of cellular damage and renal dysfunction [17, 18]. The pathological effects of ONOO^−^ and its decomposition product, ∙OH, contribute to the antioxidant depletion, alterations of the protein structure and function by tyrosine nitration, and oxidative damage observed in human diseases and animal models of diseases [19–23].

 The protective effect of (−)-epicatechin 3-*O*-gallate against ONOO^−^-mediated damage was examined using an animal model and cell culture system. This study was also carried out to elucidate whether the effect of (−)-epicatechin 3-*O*-gallate is distinct from that of several well-known free radical inhibitors, the ONOO^−^ inhibitors ebselen and uric acid, O_2_^−^ scavenger copper zinc superoxide dismutase (CuZnSOD), and the selective inducible NO synthase (iNOS) inhibitor l-*N* ^6^-(1-iminoethyl)lysine hydrochloride. To generate ONOO^−^, male Wistar rats (10-week-old, male) were subjected to ischemia-reperfusion (occlusion of the renal artery and vein with clamps) together with lipopolysaccharide (LPS) injection.

 In this study, the significant stimulation of NO and O_2_^−^ generation in response to the LPS injection plus ischemia-reperfusion process declined markedly after treatment with l-*N* ^6^-(1-iminoethyl)lysine hydrochloride and CuZnSOD, respectively (Table 1). (−)-Epicatechin 3-*O*-gallate, however, did not reverse the elevations in the plasma NO and O_2_^−^ levels resulting from LPS plus ischemia-reperfusion. This suggests that (−)-epicatechin 3-*O*-gallate does not act as a scavenger of the ONOO^−^ precursors NO and O_2_^−^. In light of these results, we hypothesized that the protective activity of (−)-epicatechin 3-*O*-gallate against ONOO^−^ could be attributed to the direct scavenging of ONOO^−^, and so we evaluated the levels of 3-nitrotyrosine and myeloperoxidase (MPO) activity as indicators of ONOO^−^ formation.

**Figure 1:**
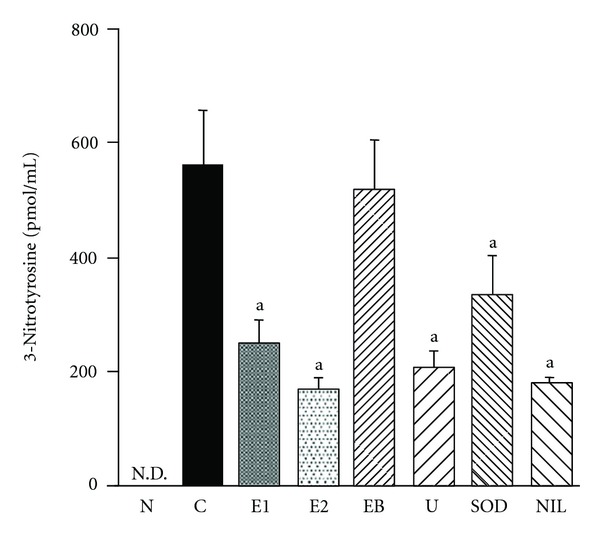
Effect of (−)-epicatechin 3-*O*-gallate and free radical inhibitors on plasma 3-nitrotyrosine level in rats. N, sham operation; C, LPS plus ischemia-reperfusion; E1, LPS plus ischemia-reperfusion after (−)-epicatechin 3-*O*-gallate (10 mg/kg body weight); E2, LPS plus ischemia-reperfusion after (−)-epicatechin 3-*O*-gallate (20 mg/kg body weight); EB, LPS plus ischemia-reperfusion after ebselen (5 mg/kg body weight); U, LPS plus ischemia-reperfusion after uric acid (62.5 mg/kg body weight); SOD, LPS plus ischemia-reperfusion after CuZnSOD (10,000 U/kg body weight); NIL, LPS plus ischemia-reperfusion after l-*N* ^6^-(1-iminoethyl)lysine hydrochloride (3 mg/kg body weight). N.D., not detectable. Significance: ^a^ *P* < 0.001 versus LPS plus ischemia-reperfused control values.

**Figure 2:**
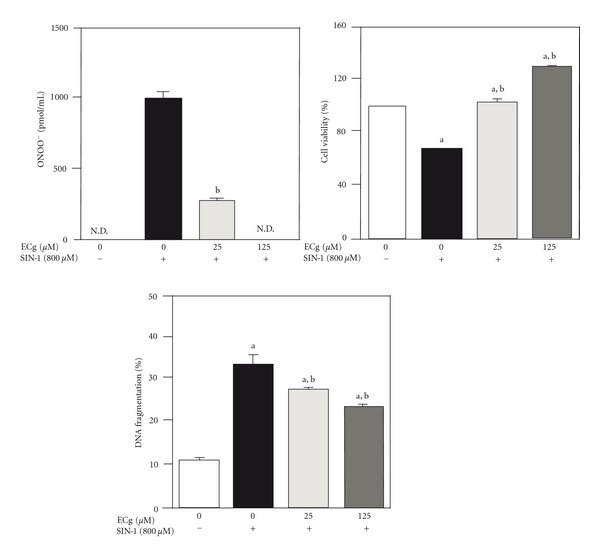
Effect of (−)-epicatechin 3-*O*-gallate on SIN-1-induced ONOO^−^ formation, viability, and DNA fragmentation in renal epithelial cells, LLC-PK_1_. N.D., not detectable. Significance: ^a^ *P* < 0.001 versus no treatment values; ^b^ *P* < 0.001 versus SIN-1 treatment values.

**Figure 3:**
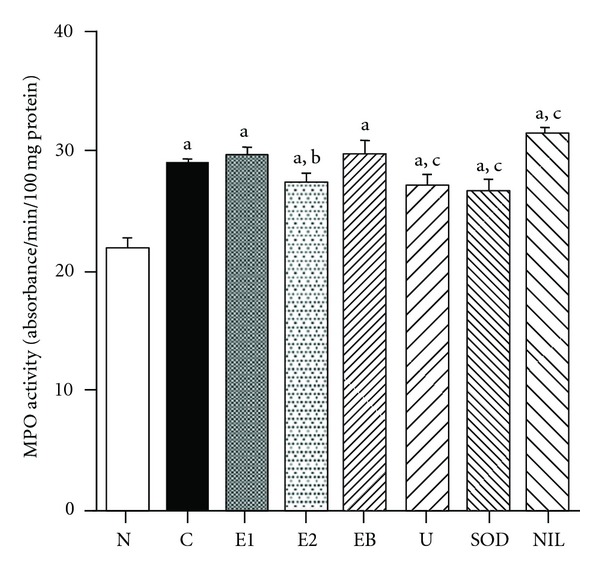
Effect of (−)-epicatechin 3-*O*-gallate and free radical inhibitors on renal MPO activity in rats. N, sham operation; C, LPS plus ischemia-reperfusion; E1, LPS plus ischemia-reperfusion after (−)-epicatechin 3-*O*-gallate (10 mg/kg body weight); E2, LPS plus ischemia-reperfusion after (−)-epicatechin 3-*O*-gallate (20 mg/kg body weight); EB, LPS plus ischemia-reperfusion after ebselen (5 mg/kg body weight); U, LPS plus ischemia-reperfusion after uric acid (62.5 mg/kg body weight); SOD, LPS plus ischemia-reperfusion after CuZnSOD (10,000 U/kg body weight); NIL, LPS plus ischemia-reperfusion after l-*N* ^6^-(1-iminoethyl)lysine hydrochloride (3 mg/kg body weight). Significance: ^a^ *P* < 0.001 versus sham operation values; ^b^ *P* < 0.01, ^c^ *P* < 0.001 versus LPS plus ischemia-reperfused control values.

 The LPS plus ischemia-reperfusion process led to elevation of the plasma 3-nitrotyrosine level in rats, suggesting that oxidative damage due to the formation of ONOO^−^ had occurred (Figure 1) and the cellular formation of ONOO^−^ increased by 3-morpholinosydnonimine (SIN-1) treatment (Figure 2). However, (−)-epicatechin 3-*O*-gallate reduced nitrotyrosine formation markedly in a dose-dependent manner compared with ebselen and CuZnSOD. The activity of (−)-epicatechin 3-*O*-gallate was comparable with that of l-*N* ^6^-(1-iminoethyl)lysine hydrochloride, although (−)-epicatechin 3-*O*-gallate did not scavenge NO (Figure 1 and Table 1). The magnitudes of the significant elevations of ONOO^−^ production in the cellular system were decreased by (−)-epicatechin 3-*O*-gallate treatment (Figure 2). Taken together, these findings indicate that (−)-epicatechin 3-*O*-gallate scavenges ONOO^−^ directly but not its precursors NO and O_2_^−^. In addition, the elevation of MPO activity was reversed by the administration of (−)-epicatechin 3-*O*-gallate, uric acid, and SOD but not by that of l-*N* ^6^-(1-iminoethyl)lysine hydrochloride (Figure 3). We consider that the reduction of MPO activity by (−)-epicatechin 3-*O*-gallate ameliorated ONOO^−^-induced oxidative damage by inhibiting protein nitration and lipid peroxidation through a mechanism distinct from that of l-*N* ^6^-(1-iminoethyl)lysine hydrochloride, which actually increased MPO activity. In addition, uric acid acted in a similar way to (−)-epicatechin 3-*O*-gallate as a direct scavenger of ONOO^−^ through the inhibition of 3-nitrotyrosine and MPO activity, and not as a scavenger of ONOO^−^ precursors (Figures 1 and 3).

The antioxidative defense system was significantly suppressed by the excessive increase of ONOO^−^ resulting from the LPS plus ischemia-reperfusion process. The administration of (−)-epicatechin 3-*O*-gallate resulted in concentration-dependent elevations of the activities of the antioxidative enzymes, SOD, catalase, and glutathione peroxidase (GSH-Px), and the cellular antioxidant reduced glutathione (GSH) (Tables 2 and 3). Furthermore, the excessive ONOO^−^ increased lipid peroxidation of renal mitochondria (Table 3), and we confirmed the mitochondrial oxidative damage caused by ONOO^−^. In contrast, the administration of (−)-epicatechin 3-*O*-gallate reduced the magnitude of the lipid peroxidation level elevation caused by the experimental process (Table 3).

 Since ONOO^−^ decomposes to form a strong and reactive oxidant, ∙OH, the effects of free radical scavengers and (−)-epicatechin 3-*O*-gallate on ∙OH also have to be evaluated to compare their protective actions against ONOO^−^. In this study, we used the spin-trap method to determine the level of ∙OH in rat renal tissue formed by the Fenton reaction, and found that the magnitude of the increase in the height of the DMPO-OH peak of rats that underwent LPS plus ischemia-reperfusion was reduced by treatment with (−)-epicatechin 3-*O*-gallate, CuZnSOD, and l-*N* ^6^-(1-iminoethyl)lysine hydrochloride (Table 4). These findings indicate that the effect of (−)-epicatechin 3-*O*-gallate on the highly reactive radical ∙OH plays a crucial role in its protective action against ONOO^−^-induced oxidative damage. Furthermore, the effects of (−)-epicatechin 3-*O*-gallate on ONOO^−^ and ∙OH were stronger than those of the other well-known free radical inhibitors tested, which can also be regarded as a mechanism distinct from that of the others. 

 The LPS plus ischemia-reperfusion process resulted in a significant elevation of the uric acid level, indicating that a pathological condition in the kidney had developed (Table 5). However, the administration of (−)-epicatechin 3-*O*-gallate reduced the uric acid level, while the other free radical inhibitors did not (Table 5). This effect of (−)-epicatechin 3-*O*-gallate on excessive uric acid levels is also considered to be a property distinct from the other free radical scavengers. The renal function parameters of serum urea nitrogen and creatinine (Cr) levels were elevated markedly by LPS plus ischemia-reperfusion, while the administration of (−)-epicatechin 3-*O*-gallate reduced these levels significantly, indicating the amelioration of renal dysfunction by (−)-epicatechin 3-*O*-gallate. In addition, uric acid and l-*N* ^6^-(1-iminoethyl)lysine hydrochloride protected against renal dysfunction induced by this process, although their activity was relatively low compared with (−)-epicatechin 3-*O*-gallate.

**Figure 4:**
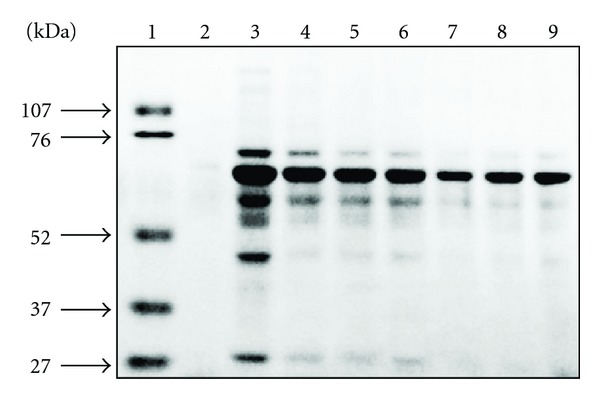
Effect of (−)-epicatechin 3-*O*-gallate and free radical inhibitors on SDS-PAGE pattern of proteinuria in rats. 1, marker; 2, sham operation; 3, LPS plus ischemia-reperfusion; 4, LPS plus ischemia-reperfusion after ebselen (5 mg/kg body weight); 5, LPS plus ischemia-reperfusion after uric acid (62.5 mg/kg body weight); 6, LPS plus ischemia-reperfusion after CuZnSOD (10,000 U/kg body weight); 7, LPS plus ischemia-reperfusion after l-*N* ^6^-(1-iminoethyl)lysine hydrochloride (3 mg/kg body weight); 8, LPS plus ischemia-reperfusion after (−)-epicatechin 3-*O*-gallate (20 mg/kg body weight); 9, LPS plus ischemia-reperfusion after (−)-epicatechin 3-*O*-gallate (10 mg/kg body weight). Markers (kDa): 107, phosphorylase B; 76, bovine serum albumin; 52, ovalbumin; 37, carbonic anhydrase; 27, soybean trypsin inhibitor.

 Our results in rats showed that the LPS plus ischemia-reperfusion process led to proteinuria, demonstrated by the sodium dodecyl sulfate-polyacrylamide gel electrophoresis (SDS-PAGE) pattern with an abundance of low- and high-molecular-weight proteins relative to the marker albumin (76 kDa) (Figure 4). The administration of (−)-epicatechin 3-*O*-gallate and l-*N* ^6^-(1-iminoethyl)lysine hydrochloride reduced the intensity of the low- and high-molecular-weight protein bands to a greater extent than the other radical inhibitors, which suggests that (−)-epicatechin 3-*O*-gallate would ameliorate proteinuria due to renal failure caused by ONOO^−^-induced oxidative damage.

In the LPS plus ischemia-reperfusion rat model, (−)-epicatechin 3-*O*-gallate, l-*N* ^6^-(1-iminoethyl)lysine hydrochloride, and uric acid showed a strong protective effect against ONOO^−^-induced oxidative damage, while CuZnSOD and ebselen exerted relatively low activity. In light of the results of this study, we suggest that the activity of (−)-epicatechin 3-*O*-gallate is distinct from that of the other free radical inhibitors, especially l-*N* ^6^-(1-iminoethyl)lysine hydrochloride and uric acid. (−)-Epicatechin 3-*O*-gallate scavenged ONOO^−^ directly, but it did not scavenge its precursors O_2_^−^ and NO. Furthermore, (−)-epicatechin 3-*O*-gallate indirectly inhibits the generation of ONOO^−^ through the enhancement of antioxidant enzyme activities. In addition, the inhibition of MPO activity by (−)-epicatechin 3-*O*-gallate would contribute to the effective inhibition of protein nitration and lipid peroxidation. (−)-Epicatechin 3-*O*-gallate was also a stronger scavenger of the ONOO^−^ decomposition product ∙OH than any of the other free radical inhibitors tested. The improvement by (−)-epicatechin 3-*O*-gallate of the renal dysfunction caused by ONOO^−^-related oxidative damage was marked and distinct from that induced by any of the other free radical inhibitors.

## 3. (−)-Epigallocatechin 3-*O*-Gallate and Adenine-Induced Renal Failure

Methylguanidine (MG) is widely recognized as a strong uremic toxin [24]. The ∙OH radical specifically plays an important role in the pathway of MG production from Cr [25]. In this study, we investigated whether the oral administration of (−)-epigallocatechin 3-*O*-gallate suppresses MG production in rats with chronic renal failure after intraperitoneal Cr injection.

**Figure 5:**
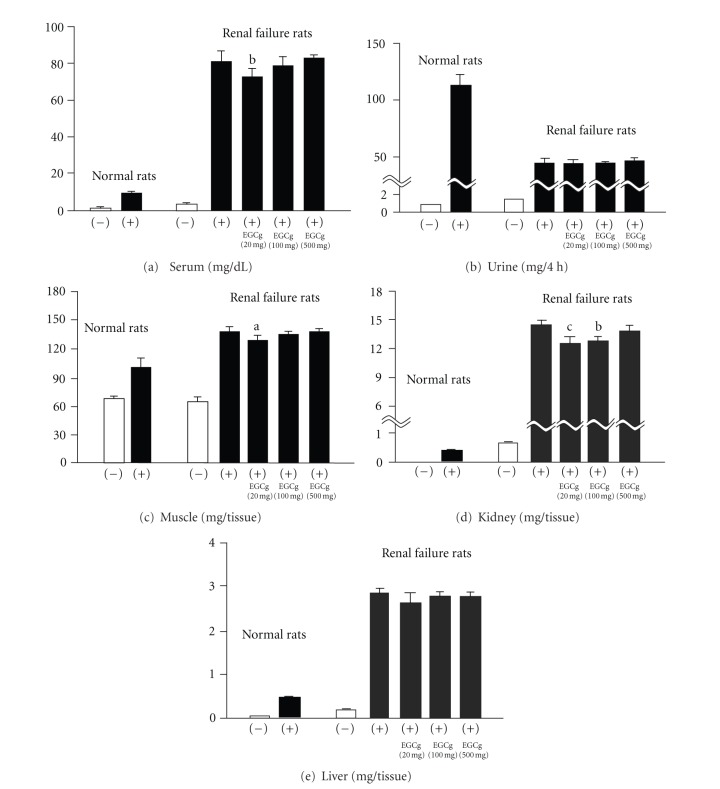
Cr levels in serum (a), urine (b), muscle (c), kidney (d), and liver (e). (−), without Cr loading; (+), with Cr loading. Significance: ^a^ *P* < 0.05, ^b^ *P* < 0.01, ^c^ *P* < 0.001 versus renal failure control rats with Cr loading.

**Figure 6:**
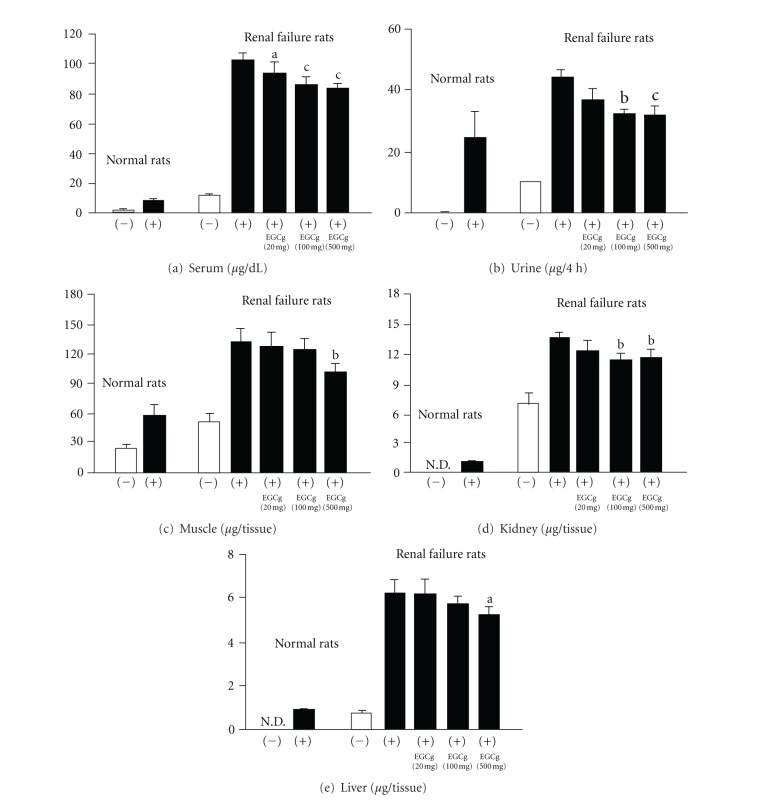
MG levels in serum (a), urine (b), muscle (c), kidney (d), and liver (e). (−), without Cr loading; (+), with Cr loading. Significance: ^a^ *P* < 0.05, ^b^ *P* < 0.01, ^c^ *P* < 0.001 versus renal failure control rats with Cr loading.

 In 10-week-old male normal rats, Cr was rapidly excreted into the urine after Cr loading, whereas, in age-matched rats with renal failure, urinary Cr excretion was low, and high levels of Cr were present in the serum, muscle, kidney, and liver, suggesting that the body was susceptible to oxidative alterations (Figure 5). After Cr loading, the MG levels in the serum, urine, muscle, liver, and kidney of rats with renal failure were higher than those of normal rats, confirming that MG production from Cr was increased in rats with renal failure (Figure 6). The oral administration of (−)-epigallocatechin 3-*O*-gallate dose-dependently reduced the serum MG levels, showing that (−)-epigallocatechin 3-*O*-gallate effectively inhibited increased MG production in which oxidative reactions markedly participate. (−)-Epigallocatechin 3-*O*-gallate (20 mg/kg body weight) reduced the urinary and kidney MG levels, which were reduced further and significantly in the 100 and 500 mg treated groups. In the muscle and liver, a significant reduction was only observed in the high dose-treated group (500 mg) (Figure 6).

 We have already demonstrated that green tea polyphenols (daily dose, 400 mg) administered for 6 months to 50 patients on dialysis decreased the blood levels of MG [26], and that concomitant treatment with green tea polyphenols during 25-day adenine-feeding periods produced a dose-dependent decrease in the serum MG level [27]. Furthermore, we reported that concomitant treatment with green tea polyphenols had protective effects against the increased serum Cr and urinary protein levels and the decreased creatinine clearance (Ccr) [7, 28], indicating that green tea polyphenols can delay deterioration of the renal function. Taking the evidence from previous and present studies into consideration, we propose that green tea polyphenols exert an MG-lowering effect in dialysis patients and rats with adenine-induced renal failure through, at least in part, two actions: the improvement of renal dysfunction, and inhibition of MG production from Cr due to their ability to scavenge ∙OH.

## 4. (−)-Epigallocatechin 3-*O*-gallate and Diabetic Nephropathy

The pathogenesis of diabetic nephropathy has been extensively discussed for many years, and it has been accepted that oxidative stress is closely involved as a causative factor stemming from persistent hyperglycemia [29, 30]. Within the diabetic kidney, glucose-dependent pathways such as increasing oxidative stress, polyol formation, and advanced glycation endproduct (AGE) accumulation, are activated.

 To evaluate the effect of (−)-epigallocatechin 3-*O*-gallate as a representative polyphenol on diabetic nephropathy, rats (10-week-old, male) with subtotal nephrectomy plus streptozotocin injection were orally administered (−)-epigallocatechin 3-*O*-gallate at doses of 25, 50, and 100 mg/kg body weight/day for 50 days.

 Hyperglycemia is the principle factor responsible for structural alterations at the renal level, and The Diabetes Control and Complications Trial Research Group [31] has elucidated that hyperglycemia is directly linked to diabetic microvascular complications, particularly in the kidney; therefore, glycemic control remains the main target of therapy. In this study, the glucose level of diabetic nephropathy rats showed a significant approximately 3-fold increase; however, (−)-epigallocatechin 3-*O*-gallate inhibited this increase dose-dependently (Table 6). In addition, the typical patterns of serum constituents, that is, a decrease in total protein and albumin due to their excessive excretion via urine, and also an increase in lipids, for example, total cholesterol and triglycerides, whose abnormal metabolism has been proven to play a role in the pathogenesis of diabetic nephropathy [32] and to enhance lipid peroxidation, were all improved by the administration of (−)-epigallocatechin 3-*O*-gallate (Table 6). Therefore, we suggest that (−)-epigallocatechin 3-*O*-gallate had a positive effect on serum glucose and lipid metabolic abnormalities.

**Figure 7:**
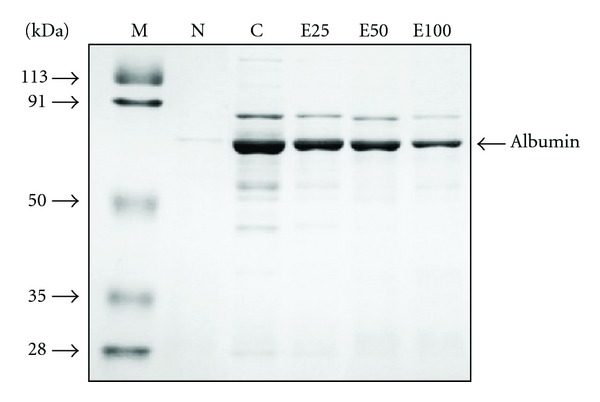
SDS-PAGE pattern of proteinuria in normal rats (N) and diabetic nephrectomized rats treated with (−)-epigallocatechin 3-*O*-gallate at 25 mg/kg body weight/day (E25), 50 mg/kg body weight/day (E50), 100 mg/kg body weight/day (E100), or water (control, C) for 50 days. Lane M shows the molecular weight marker.

 The results of the study presented here demonstrate that diabetic nephropathy rats showed significant increases in the serum urea nitrogen, Cr, and urinary protein excretion rate, whereas the Ccr level showed a significant decrease compared with normal rats, representing a decline in the renal function (Table 7). However, the (−)-epigallocatechin 3-*O*-gallate treatment positively affected these parameters, especially in the group given 100 mg (Table 7). For further investigation, we performed pattern analysis of proteinuria using SDS-PAGE, and the (−)-epigallocatechin 3-*O*-gallate treatment led to a clear decrease at all parts of the molecule (Figure 7). These data suggest that not only the improvement of proteinuria, but also its individual fractions, may, at least in part, ameliorate the development of glomerular and tubulointerstitial injury.

**Figure 8:**
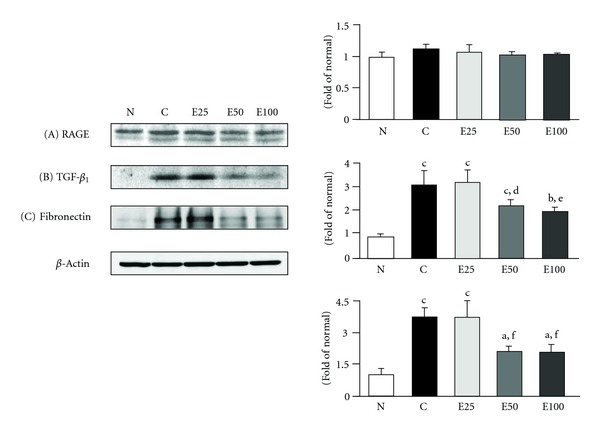
Western blot analyses of RAGE (A), TGF-*β*_1_ (B), and fibronectin (C) protein expression in the renal cortex of normal rats (N) and diabetic nephrectomized rats treated with (−)-epigallocatechin 3-*O*-gallate at 25 mg/kg body weight/day (E25), 50 mg/kg body weight/day (E50), 100 mg/kg body weight/day (E100), or water (control, C) for 50 days. Significance: ^a^ *P* < 0.05, ^b^ *P* < 0.01, ^c^ *P* < 0.001 versus normal values; ^d^ *P* < 0.05, ^e^ *P* < 0.01, ^f^ *P* < 0.001 versus diabetic nephropathy control values.

**Figure 9:**
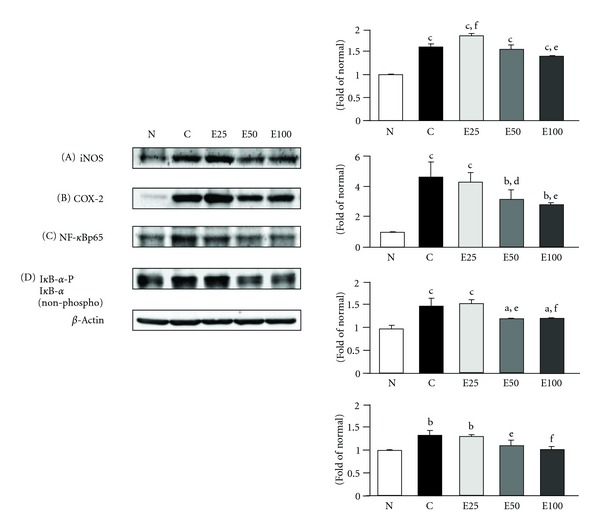
Western blot analyses of iNOS (A), COX-2 (B), NF-*κ*Bp65 (C), and I*κ*B-*α* (phosphorylated and nonphosphorylated) (D) protein expression in the renal cortex of normal rats (N) and diabetic nephrectomized rats treated with (−)-epigallocatechin 3-*O*-gallate at 25 mg/kg body weight/day (E25), 50 mg/kg body weight/day (E50), 100 mg/kg body weight/day (E100), or water (control, C) for 50 days. Significance: ^a^ *P* < 0.05, ^b^ *P* < 0.01, ^c^ *P* < 0.001 versus normal values; ^d^ *P* < 0.05, ^e^ *P* < 0.01, ^f^ *P* < 0.001 versus diabetic nephropathy control values.

 In the state of diabetic nephropathy, there is increased glomerular basement membrane thickening and mesangial extracellular matrix (ECM) deposition, followed by mesangial hypertrophy and diffuse and nodular glomerular sclerosis, and these structural changes may be directly influenced by AGEs through excessive cross-linking of the matrix molecules in a receptor-independent way [33, 34]. In this study, we demonstrated that renal AGE accumulation observed in diabetic nephropathy rats was decreased by (−)-epigallocatechin 3-*O*-gallate administration, although (−)-epigallocatechin 3-*O*-gallate showed only a slight tendency to reduce renal receptor for advanced glycation endproduct (RAGE) expression in diabetic nephropathy rats (Figure 8). However, a marked antioxidative activity of renal tissue was shown in the level of lipid peroxidation at 50 and 100 mg doses of (−)-epigallocatechin 3-*O*-gallate, resembling the results of iNOS, cyclooxygenase (COX)-2, nuclear factor-*κ*B (NF-*κ*B), and phosphorylated inhibitor binding protein *κ*B-*α* (I*κ*B-*α*) (Figure 9), and the fibrogenic cytokine transforming growth factor (TGF)-*β*_1_ and fibronectin protein expression in the renal cortex (Figure 8).

**Figure 10:**
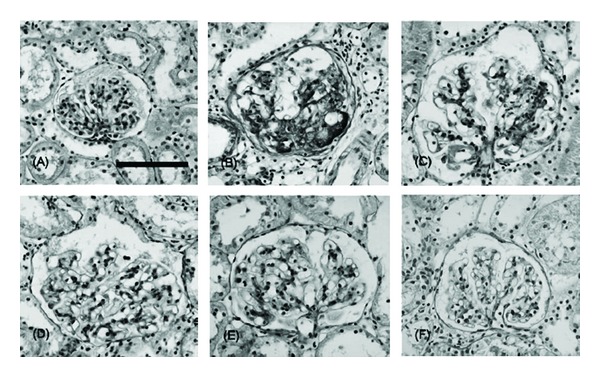
Photomicrographs of the glomeruli in normal rats (A) and diabetic nephrectomized rats treated with (−)-epigallocatechin 3-*O*-gallate at 25 mg/kg body weight/day (D), 50 mg/kg body weight/day (E), 100 mg/kg body weight/day (F), or water (control, B and C) for 50 days. Scale bar, 100 *μ*m.

 Moreover, diabetic nephropathy rats used in the present study showed significant glomerular hypertrophy and diffuse and exudative lesions. Longitudinal hyperfiltration is associated with renal enlargement such as an increase in the glomerular size, and diffuse lesion development is dependent on increased mesangial matrix and glomerular basement membrane thickening, because both are composed of ECM molecules, as in the case of the TGF-*β* system, and they also correlate with proteinuria. The other phenomenon, the exudative lesion called the capsular drop and fibrin cap, is suggested to consist of plasma components such as IgM, fibrinogen, and AGEs. According to the results of histopathological evaluation, although diabetic nephropathy rats showed a 2.2-fold increase in the glomerular area, mild but significant increases in diffuse and exudative lesions, and a slight increase in the mesangial matrix, (−)-epigallocatechin 3-*O*-gallate could affect glomerular hypertrophy and these lesions at 50 and 100 mg doses, reflecting the effects of AGEs, TGF-*β*_1_, and fibronectin levels (Figures 8 and 10). Hence, we may hypothesize that (−)-epigallocatechin 3-*O*-gallate could be advantageous against diabetic kidney damage, which correlates with AGEs with or without a receptor-dependent pathway and their related inflammatory responses, and then (−)-epigallocatechin 3-*O*-gallate subsequently suppresses the induction of mesangial hypertrophy and fibronectin synthesis in diabetic nephropathy.

 Our observations presented here suggest that (−)-epigallocatechin 3-*O*-gallate has a beneficial effect on diabetic nephropathy via suppressing hyperglycemia, AGEs, their related oxidative stress and cytokine activations, and also pathological states due to its synergistic effect. This study may provide original and strong supporting evidence for the efficacy of (−)-epigallocatechin 3-*O*-gallate in the early stage of diabetic nephropathy, suggesting that it would be a superior aid for the management of patients with diabetic nephropathy.

## 5. Conclusion and Future Prospects

Much attention regarding green tea's benefits has been focused on the role of antioxidant activity in relation to the aging process and degenerative diseases like cancer, cardiovascular disease, and diabetes. This paper shows that, based on antioxidant activity, green tea polyphenols and their constituents exert protective effects on renal damage caused by various toxic situations such as an excessive arginine supply, strong oxidative radicals, renal toxin, diabetic nephropathy, and type 2 diabetes. Therefore, we expect that green tea polyphenols have the potential to prevent organ failure and, in particular, provide a promising therapeutic approach to renal disorders. As green tea is already one of the most popular beverages worldwide, its role should be understandably elucidated in the direct and indirect prevention of chronic diseases. In order to explain the potential mechanisms of green tea polyphenols for protection against organ damage concomitant with chronic disease, additional research is needed on the pharmacokinetics of tea constituents as well as exploration at the cellular level. Furthermore, well-designed observational epidemiological studies and intervention trials will generate clear and safe conclusions concerning the protective effects of tea.

## Figures and Tables

**Table 1:** Effect of (−)-epicatechin 3-*O*-gallate and free radical inhibitors on plasma NO and O_2_^−^ radicals in rats.

Group	NO (*μ*M)	O_2_^−^ (O.D.)
Sham operation	1.71 ± 0.18	0.315 ± 0.013
LPS plus ischemia-reperfusion		
Control	15.33 ± 0.72^b^	0.371 ± 0.011^a^
(−)-Epicatechin 3-*O*-gallate (10 mg/kg B.W.)	15.02 ± 1.15^b^	0.377 ± 0.019^b^
(−)-Epicatechin 3-*O*-gallate (20 mg/kg B.W.)	14.24 ± 0.33^b^	0.401 ± 0.008^b^
Ebselen (5 mg/kg B.W.)	15.98 ± 1.35^b^	0.345 ± 0.007
Uric acid (62.5 mg/kg B.W.)	15.08 ± 1.15^b^	0.360 ± 0.026^a^
SOD (10,000 U/kg B.W.)	19.04 ± 1.72^b,d^	0.336 ± 0.016^c^
l-*N* ^6^-(1-iminoethyl)lysine hydrochloride (3 mg/kg B.W.)	3.39 ± 0.25^e^	0.363 ± 0.022^a^

Significance: ^a^ *P* < 0.01, ^b^ *P* < 0.001 versus sham operation values; ^c^ *P* < 0.05, ^d^ *P* < 0.01, ^e^ *P* < 0.001 versus LPS plus ischemia-reperfused control values.

**Table 2:** Effect of (−)-epicatechin 3*-O-*gallate on oxygen species-scavenging enzymes in renal tissue.

Group	SOD	Catalase	GSH-Px
	(U/mg protein)	(U/mg protein)	(U/mg protein)
Sham operation	31.82 ± 2.29	255.3 ± 35.0	138.7 ± 10.3
LPS plus ischemia-reperfusion			
Control	16.67 ± 2.52^c^	146.8 ± 19.3^c^	79.5 ± 7.2^c^
(−)-Epicatechin 3*-O-*gallate (10 *μ*moles/kg B.W./day)	18.18 ± 1.70^c^	176.0 ± 15.3^c^	105.7 ± 8.0^c,e^
(−)-Epicatechin 3*-O-*gallate (20 *μ*moles/kg B.W./day)	21.45 ± 3.67^c,d^	194.4 ± 22.6^b,d^	118.7 ± 11.0^a,f^

Significance: ^a^ *P* < 0.05, ^b^ *P* < 0.01, ^c^ *P* < 0.001 versus sham operation values; ^d^ *P* < 0.05, ^e^ *P* < 0.01, ^f^ *P* < 0.001 versus LPS plus ischemia-reperfused control values.

**Table 3:** Effect of (−)-epicatechin 3-*O*-gallate on the oxidative damage of renal mitochondria.

Group	GSH	TBA-reactive substance
	(nmol/mg protein)	(nmol/mg protein)
Sham operation	4.42 ± 0.09	0.121 ± 0.001
LPS plus ischemia-reperfusion		
Control	2.75 ± 0.14^a^	0.165 ± 0.007^a^
(−)-Epicatechin 3-*O*-gallate (10 *μ*moles/kg B.W./day)	3.72 ± 0.18^a,b^	0.147 ± 0.003^a,b^
(−)-Epicatechin 3-*O*-gallate (20 *μ*moles/kg B.W./day)	3.77 ± 0.21^a,b^	0.144 ± 0.007^a,b^

Significance: ^a^ *P* < 0.001 versus sham operation values; ^b^ *P* < 0.001 versus LPS plus ischemia-reperfused control values.

**Table 4:** Effect of (−)-epicatechin 3-*O*-gallate and free radical inhibitors on renal hydroxyl radical in rats.

Group	Hydroxyl radical (DMPO-OH)
Sham operation	0.29 ± 0.07
LPS plus ischemia-reperfusion	
Control	1.15 ± 0.15^a^
(−)-Epicatechin 3-*O*-gallate (10 mg/kg B.W.)	0.18 ± 0.01^b^
(−)-Epicatechin 3-*O*-gallate (20 mg/kg B.W.)	0.17 ± 0.01^b^
Ebselen (5 mg/kg B.W.)	1.10 ± 0.18^a^
Uric acid (62.5 mg/kg B.W.)	1.06 ± 0.07^a^
SOD (10,000 U/kg B.W.)	0.22 ± 0.01^b^
l-*N* ^6^-(1-iminoethyl)lysine hydrochloride (3 mg/kg B.W.)	0.20 ± 0.03^b^

Significance: ^a^ *P* < 0.001 versus sham operation values; ^b^ *P* < 0.001 versus LPS plus ischemia-reperfused control values.

**Table 5:** Effect of (−)-epicatechin 3-*O*-gallate and free radical inhibitors on plasma uric acid level in rats.

Group	Uric acid (mg/dL)
Sham operation	1.53 ± 0.18
LPS plus ischemia-reperfusion	
Control	1.95 ± 0.03^a^
(−)-Epicatechin 3-*O*-gallate (10 mg/kg B.W.)	1.64 ± 0.24
(−)-Epicatechin 3-*O*-gallate (20 mg/kg B.W.)	1.12 ± 0.11^c^
Ebselen (5 mg/kg B.W.)	2.15 ± 0.37^b^
Uric acid (62.5 mg/kg B.W.)	1.96 ± 0.35
SOD (10,000 U/kg B.W.)	2.09 ± 0.09^a^
l-*N* ^6^-(1-iminoethyl)lysine hydrochloride (3 mg/kg B.W.)	1.57 ± 0.25

Significance: ^a^ *P* < 0.05, ^b^ *P* < 0.01 versus sham operation values; ^c^ *P* < 0.001 versus LPS plus ischemia-reperfused control values.

**Table 6:** Serum constituents at 50 days of administration.

Items	Normal	Control	(−)-Epigallocatechin 3-*O*-gallate		
			25 mg/kg B.W./day	50 mg/kg B.W./day	100 mg/kg B.W./day
Glucose (mg/dL)	193 ± 9	592 ± 38^c^	497 ± 22^c,e^	487 ± 22^c,e^	460 ± 19^c,e^
Total protein (g/dL)	4.75 ± 0.11	4.21 ± 0.08^c^	4.20 ± 0.10^c^	4.37 ± 0.07^c,d^	4.44 ± 0.06^c,e^
Albumin (g/dL)	2.88 ± 0.04	2.38 ± 0.08^c^	2.43 ± 0.06^c^	2.56 ± 0.06^c,e^	2.62 ± 0.05^c,e^
Total cholesterol (mg/dL)	46.4 ± 2.4	113.6 ± 12.7^c^	102.3 ± 6.0^c^	83.3 ± 6.4^c,e^	77.7 ± 6.8^c,e^
Triglycerides (mg/dL)	63.7 ± 6.3	143.1 ± 31.4^c^	126.6 ± 15.7^a^	120.9 ± 27.3^a^	116.6 ± 26.3^a^
TBA-reactive substance (nmol/mL)	1.56 ± 0.08	3.70 ± 0.39^c^	2.48 ± 0.18^b,e^	2.50 ± 0.34^b,e^	2.16 ± 0.24^e^

Significance: ^a^ *P* < 0.05, ^b^ *P* < 0.01, ^c^ *P* < 0.001 versus normal values; ^d^ *P* < 0.05, ^e^ *P* < 0.001 versus diabetic nephropathy control values.

**Table 7:** Renal functional parameters at 50 days of administration.

Items	Normal	Control	(−)-Epigallocatechin 3-*O*-gallate		
			25 mg/kg B.W./day	50 mg/kg B.W./day	100 mg/kg B.W./day
Serum urea nitrogen (mg/dL)	16.8 ± 0.5	44.5 ± 3.1^b^	37.9 ± 1.8^b,d^	38.0 ± 2.6^b,d^	28.8 ± 1.4^b,d^
Serum Cr (mg/dL)	0.38 ± 0.01	0.94 ± 0.09^b^	0.90 ± 0.08^b^	0.82 ± 0.06^b^	0.66 ± 0.05^b,d^
Ccr (ml/kg B.W./min)	7.20 ± 0.26	3.35 ± 0.43^b^	3.41 ± 0.32^b^	3.65 ± 0.37^b^	4.09 ± 0.35^b,c^
Urinary protein (mg/day)	19.1 ± 0.7	82.3 ± 13.3^b^	64.0 ± 11.9^b^	47.9 ± 14.6^a,d^	40.6 ± 6.4^d^

Significance: ^a^ *P* < 0.05, ^b^ *P* < 0.001 versus normal values; ^c^ *P* < 0.05, ^d^ *P* < 0.001 versus diabetic nephropathy control values.

## References

[1] Fiorillo C, Oliviero C, Rizzuti G, Nediani C, Pacini A, Nassi P (1998). Oxidative stress and antioxidant defenses in renal patients receiving regular haemodialysis. *Clinical Chemistry and Laboratory Medicine*.

[2] Handelman GJ, Walter MF, Adhikarla R (2001). Elevated plasma F2-isoprostanes in patients on long-term hemodialysis. *Kidney International*.

[3] Kakimoto M, Inoguchi T, Sonta T (2002). Accumulation of 8-hydroxy-2′-deoxyguanosine and mitochondrial DNA deletion in kidney of diabetic rats. *Diabetes*.

[4] Yokozawa T, Chen CP, Rhyu DY, Tanaka T, Park JC, Kitani K (2002). Potential of sanguiin H-6 against oxidative damage in renal mitochondria and apoptosis mediated by peroxynitrite in vivo. *Nephron*.

[5] Hahn S, Krieg RJ, Hisano S (1999). Vitamin E suppresses oxidative stress and glomerulosclerosis in rat remnant kidney. *Pediatric Nephrology*.

[6] Chung HY, Yokozawa T, Soung DY, Kye IS, No JK, Baek BS (1998). Peroxynitrite-scavenging activity of green tea tannin. *Journal of Agricultural and Food Chemistry*.

[7] Yokozawa T, Dong E, Nakagawa T (1998). In vitro and in vivo studies on the radical-scavenging activity of tea. *Journal of Agricultural and Food Chemistry*.

[8] Yokozawa T, Cho EJ, Nakagawa T, Terasawa K, Takeuchi S (2000). Inhibitory effect of green tea tannin on free radical-induced injury to the renal epithelial cell line, LLC-PK_1_. *Pharmacy and Pharmacology Communications*.

[9] Nakagawa T, Yokozawa T (2002). Direct scavenging of nitric oxide and superoxide by green tea. *Food and Chemical Toxicology*.

[10] Rah DK, Han DW, Baek HS, Hyon SH, Park BY, Park JC (2007). Protection of rabbit kidney from ischemia/reperfusion injury by green tea polyphenol pretreatment. *Archives of Pharmacal Research*.

[11] Khan SA, Priyamvada S, Farooq N, Khan S, Khan MW, Yusufi ANK (2009). Protective effect of green tea extract on gentamicin-induced nephrotoxicity and oxidative damage in rat kidney. *Pharmacological Research*.

[12] Peng A, Ye T, Rakheja D (2011). The green tea polyphenol (-)-epigallocatechin-3-gallate ameliorates experimental immune-mediated glomerulonephritis. *Kidney International*.

[13] Halliwell B, Gutteridge JMC (1990). Role of free radicals and catalytic metal ions in human disease: An overview. *Methods in Enzymology*.

[14] Yokozawa T, Rhyu DY, Cho EJ (2004). (-)-Epicatechin 3-*O*-gallate ameliorates the damages related to peroxynitrite production by mechanisms distinct from those of other free radical inhibitors. *Journal of Pharmacy and Pharmacology*.

[15] Nakagawa T, Yokozawa T, Sano M, Takeuchi S, Kim M, Minamoto S (2004). Activity of (-)-epigallocatechin 3-*O*-gallate against oxidative stress in rats with adenine-induced renal failure. *Journal of Agricultural and Food Chemistry*.

[16] Yamabe N, Yokozawa T, Oya T, Kim M (2006). Therapeutic potential of (-)-epigallocatechin 3-*O*-gallate on renal damage in diabetic nephropathy model rats. *The Journal of Pharmacology and Experimental Therapeutics*.

[17] Radi R, Beckman JS, Bush KM, Freeman BA (1991). Peroxynitrite-induced membrane lipid peroxidation: The cytotoxic potential of superoxide and nitric oxide. *Archives of Biochemistry and Biophysics*.

[18] Douki T, Cadet J, Ames BN (1996). An adduct between peroxynitrite and 2'-deoxyguanosine: 4,5-dihydro-5-hydroxy-4-(nitrosooxy)-2'-deoxyguanosine. *Chemical Research in Toxicology*.

[19] Fukuyama N, Takebayashi Y, Hida M, Ishida H, Ichimori K, Nakazawa H (1997). Clinical evidence of peroxynitrite formation in chronic renal failure patients with septic shock. *Free Radical Biology and Medicine*.

[20] Ischiropoulos H (1998). Biological tyrosine nitration: a pathophysiological function of nitric oxide and reactive oxygen species. *Archives of Biochemistry and Biophysics*.

[21] Nakazawa H, Fukuyama N, Takizawa S, Tsuji C, Yoshitake M, Ishida H (2000). Nitrotyrosine formation and its role in various pathological conditions. *Free Radical Research*.

[22] Ceriello A, Mercuri F, Quagliaro L (2001). Detection of nitrotyrosine in the diabetic plasma: evidence of oxidative stress. *Diabetologia*.

[23] Cuzzocrea S, Reiter RJ (2001). Pharmacological action of melatonin in shock, inflammation and ischemia/reperfusion injury. *European Journal of Pharmacology*.

[24] Giovannetti S, Balestri PL, Barsotti G (1973). Methylguanidine in uremia. *Archives of Internal Medicine*.

[25] Ienaga K, Yokozawa T (2011). Creatinine and HMH (5-hydroxy-1-methylhydantoin, NZ-419) as intrinsic hydroxyl radical scavengers. *Drug Discoveries & Therapeutics*.

[26] Yokozawa T, Oura H, Shibata T (1996.). Effects of green tea tannin in dialysis patients. *Journal of Traditional Medicines*.

[27] Yokozawa T, Dong E, Oura H (1997). Proof that green tea tannin suppresses the increase in the blood methylguanidine level associated with renal failure. *Experimental and Toxicologic Pathology*.

[28] Yokozawa T, Chung HY, He LQ, Oura H (1996). Effectiveness of green tea tannin on rats with chronic renal failure. *Bioscience, Biotechnology and Biochemistry*.

[29] Baynes JW, Thorpe SR (1999). Role of oxidative stress in diabetic complications: a new perspective on an old paradigm. *Diabetes*.

[30] Ha H, Kim KH (1999). Pathogenesis of diabetic nephropathy: the role of oxidative stress and protein kinase C. *Diabetes Research and Clinical Practice*.

[31] The Diabetes Control and Complications Trial Research Group (1993). The effect of intensive treatment of diabetes on the development and progression of long-term complications in insulin-dependent diabetes mellitus. *The New England Journal of Medicine*.

[32] Sun L, Halaihel N, Zhang W, Rogers T, Levi M (2002). Role of sterol regulatory element-binding protein 1 in regulation of renal lipid metabolism and glomerulosclerosis in diabetes mellitus. *The Journal of Biological Chemistry*.

[33] Vlassara H, Fuh H, Makita Z, Krungkrai S, Cerami A, Bucala R (1992). Exogenous advanced glycosylation end products induce complex vascular dysfunction in normal animals: a model for diabetic and aging complications. *Proceedings of the National Academy of Sciences of the United States of America*.

[34] Vlassara H, Striker LJ, Teichberg S, Fuh H, Li YM, Steffes M (1994). Advanced glycation end products induce glomerular sclerosis and albuminuria in normal rats. *Proceedings of the National Academy of Sciences of the United States of America*.

